# Immuno-Metabolic Reprogramming in Metabolic Syndrome and Its Cardiovascular Complications: An Integrative Bioinformatics Study

**DOI:** 10.3390/ijms27135923

**Published:** 2026-06-30

**Authors:** Komal Shrivastav, Sushama Jadhav, Pratik Mahajan, Vijay Chauware, Vijay Nema

**Affiliations:** 1 ICMR-National Institute for Tribal Health Research (NITHR), Jabalpur 482003, India; kshrivastava880@gmail.com; 2 Academy of Scientific and Innovative Research (AcSIR), Ghaziabad 201002, India; 3 ICMR-National Institute of Translational Virology and AIDS Research (NITVAR), Pune 411026, India; sushamajadhav9@gmail.com (S.J.); vijaychauware@gmail.com (V.C.); 4 Krishna Institute of Science and Technology, Krishna Vishwa Vidyapeeth (Deemed to be University), Karad 415539, India; pratik.mahajan532@gmail.com

**Keywords:** metabolic syndrome, cardiovascular diseases, coronary artery disease, type-2 diabetes mellitus, system’s biology

## Abstract

Metabolic syndrome (MeS) is a major risk factor for cardiovascular disease and is characterized by chronic low-grade inflammation, immune dysregulation, and metabolic abnormalities. However, the molecular mechanisms linking MeS to diabetic coronary artery disease (DMCAD) remain incompletely understood. Publicly available peripheral blood mononuclear cell (PBMC) transcriptomic datasets of MeS and DMCAD were analyzed using an integrative bioinformatics approach. Differentially expressed genes (DEGs) were identified using the limma package, followed by functional enrichment, protein–protein interaction (PPI) network construction, weighted gene co-expression network analysis (WGCNA), gene set enrichment analysis (GSEA), and miRNA regulatory network analysis. Candidate genes were further evaluated using an independent type 2 diabetes mellitus (T2DM) dataset for external transcriptomic validation. Integrated analyses identified immune-inflammatory and immuno-metabolic pathways as central features of both MeS and DMCAD. Enrichment analyses highlighted cytokine signaling, leukocyte activation, chemotaxis, complement activation, oxidative stress, and vascular inflammatory responses. Network analyses identified CD86, CD33, CCR1, C5AR1, FPR1, CXCL16, and LILRA5 as key hub genes associated with immune regulation and cardiometabolic dysfunction. External transcriptomic validation supported the relevance of CD33, CD86, and LILRA5. miRNA network analysis identified members of the miR-17/92 family and miR-146a-5p as potential upstream regulators. TAM 2.0 enrichment analysis further linked these miRNAs to metabolic syndrome, diabetes mellitus, atherosclerosis, coronary heart disease, immune response, inflammation, and angiogenesis. Our findings suggest that coordinated immune-inflammatory and metabolic signaling networks contribute to the progression from MeS to DMCAD. The identified hub genes and miRNAs may serve as potential biomarkers and therapeutic targets for inflammation-driven cardiometabolic disease.

## 1. Introduction

Metabolic syndrome (MeS) represents a complex and multifactorial metabolic disorder characterized by the following conditions:

1. Hypertension (consistently elevated blood pressure);
2. Insulin resistance;
3. Central obesity (abnormal accumulation of fat in the abdominal region);
4. Dyslipidemia with low HDL cholesterol and high triglycerides;
5. Impaired glucose homeostasis.

The prevalence of MeS has increased substantially worldwide and is closely associated with an increased risk of type 2 diabetes mellitus (T2DM), cardiovascular disease (CVD), and premature mortality. Sedentary lifestyles, unhealthy dietary habits, and rising obesity rates are some of the reasons for the increased prevalence. Increasing evidence suggests that chronic low-grade inflammation, immune dysregulation, oxidative stress, and altered metabolic signaling are central to development of T2DM and CVD [1]. Individuals with MeS exhibit a significantly higher risk of coronary artery disease (CAD), myocardial infarction, stroke, and heart failure compared with metabolically healthy individuals [2]. Despite considerable advances in the management of cardiometabolic disorders, the molecular mechanisms that drive progression from metabolic dysfunction to cardiovascular complications remain incompletely understood. Chronic inflammation is recognized as a major pathogenic component in several diseases, including obesity, cardiovascular diseases, and diabetes-associated complications. Recent advances in bioinformatics and systems biology have enabled integrative analysis of publicly available transcriptomic datasets to identify disease-associated genes, pathways, and regulatory networks. MeS is generally progressive in nature, with many affected individuals eventually developing type 2 diabetes mellitus (T2DM), thereby substantially increasing their risk of cardiovascular complications. The coexistence of these metabolic abnormalities promotes chronic low-grade inflammation, oxidative stress, endothelial dysfunction, and impaired glucose homeostasis, collectively contributing to the development of atherosclerosis and coronary artery disease [3].

Peripheral blood mononuclear cells (PBMCs) provide an accessible systemic representation of immune-associated transcriptional alterations occurring during chronic metabolic and inflammatory disorders. Transcriptomic profiling of PBMCs has increasingly been utilized to investigate immune-metabolic reprogramming associated with metabolic syndrome, diabetes, and cardiovascular disease progression [4]. However, despite extensive evidence supporting the contribution of inflammation to cardiometabolic diseases, the shared molecular mechanisms linking MeS with cardiovascular complications remain incompletely characterized.

In the current study, we did an integrative bioinformatics analysis of PBMC microarray datasets associated with metabolic syndrome and diabetic coronary artery disease (DMCAD). One of the main CVDs affecting people worldwide is coronary artery disease (CAD), and the comorbidity of DMCAD makes our integrated study extremely pertinent to MeS. Weighted Gene Co-expression Network Analysis (WGCNA), protein–protein interaction (PPI) network analysis, and Gene Set Enrichment Analysis (GSEA) have emerged as powerful tools for identifying biologically meaningful gene modules and signaling pathways associated with complex diseases. Furthermore, integration of microRNA (miRNA)-based regulatory networks can provide additional insights into post-transcriptional mechanisms governing immune and metabolic responses. Identification of convergent transcriptional and regulatory signatures across different cardiometabolic conditions may improve understanding of disease progression and reveal novel therapeutic targets [5]. Differentially expressed genes (DEG) analysis, functional enrichment analysis, PPI interaction network construction, hub gene identification, and miRNA regulatory analysis were performed to identify shared inflammatory and immuno-metabolic mechanisms associated with MeS and cardiovascular complications. Through external transcriptomic validation and multi-layered network analysis, the study aims to characterize inflammation-centered transcriptional alterations and identify candidate molecular regulators potentially involved in the progression of metabolic dysfunction toward coronary artery disease.

## 2. Result

### 2.1. Dataset-Level Quality Assessment and Quality Control for GSE98895 (Metabolic Syndrome)

Quality-control analyses demonstrated high consistency across all samples included in GSE98895. Density distribution plots showed highly overlapping expression profiles, indicating comparable transcriptomic distributions and the absence of major technical artifacts following normalization. Boxplot analysis further confirmed uniform expression distributions across samples.

**Figure 1:**
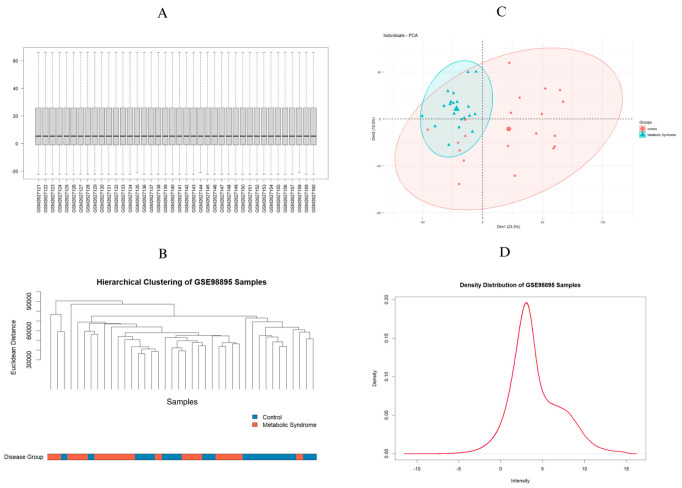
Quality-control assessment of the GSE98895 (MeS) dataset. Quality-control evaluation of the GSE98895 metabolic syndrome dataset. (**A**) Boxplot showing normalized gene-expression distributions across samples. (**B**) Hierarchical clustering dendrogram demonstrating sample similarity and absence of distinct outliers. (**C**) Principal component analysis (PCA) showing partial separation between metabolic syndrome and healthy control samples. (**D**) Density distribution plot demonstrating highly overlapping expression profiles across samples, indicating successful normalization and minimal technical variation.

Principal component analysis (PCA) revealed partial separation between metabolic syndrome and healthy control samples, with PC1 and PC2 explaining 23.3% and 10.5% of the total variance, respectively. Although biological heterogeneity was evident within both groups, no sample exceeded the predefined outlier threshold of ±3 standard deviations along either principal component. Hierarchical clustering analysis supported these findings, demonstrating coherent sample grouping without isolated or aberrantly clustered samples. Collectively, the QC analyses indicated satisfactory data quality, and all 40 samples were retained for subsequent differential expression analyses (Figure 1).

### 2.2. Dataset-Level Quality Assessment and Quality Control for GSE250283 (Diabetes Mellitus with and Without Coronary Artery Disease)

The GSE250283 study included expression data generated on two GEO platforms. GPL28098, comprising 56 peripheral blood mononuclear cell (PBMC) samples and 33,427 probes, was selected for analysis because it contained the complete study cohort, including healthy controls (*n* = 15), diabetes mellitus patients without coronary artery disease (DMnoCAD, *n* = 21), and diabetes mellitus patients with coronary artery disease (DMCAD, *n* = 20). GPL36360 contained a smaller independent subset of samples (*n* = 13) and was not integrated into the primary analysis to avoid introducing platform-specific effects.

**Figure 2:**
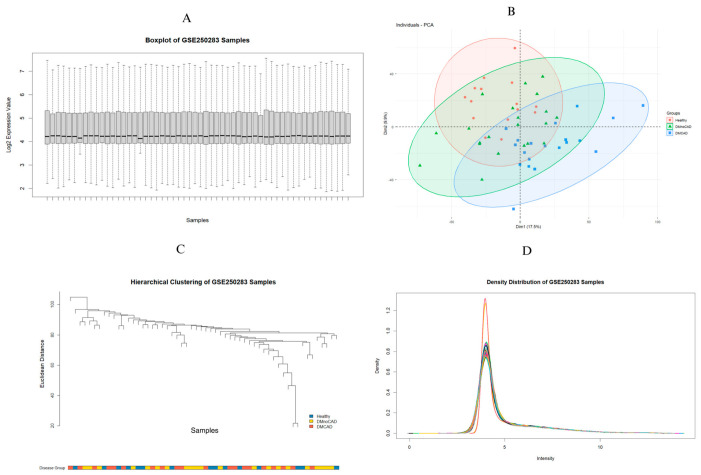
Quality-control assessment of the GSE250283 (DMCAD) dataset. Quality-control evaluation of the GSE250283 dataset containing healthy controls, diabetes mellitus without coronary artery disease (DMnoCAD), and diabetes mellitus-associated coronary artery disease (DMCAD) samples. (**A**) Boxplot of normalized expression values. (**B**) Hierarchical clustering dendrogram showing overall sample consistency. (**C**) PCA demonstrating separation among clinical groups while preserving within-group clustering. (**D**) Density distribution plot indicating comparable transcriptomic distributions across samples. Colored lines in panel (**D**) represent individual samples, with colors corresponding to the disease groups: Healthy (blue), DMnoCAD (yellow), and DMCAD (red). No samples met the criteria for exclusion following combined quality-control assessment.

Quality-control assessment of the GPL28098 dataset demonstrated overall high sample consistency. Density plots and boxplots showed comparable expression distributions across samples, indicating successful normalization and minimal technical variation. PCA revealed separation among the clinical groups while preserving within-group clustering. Outlier screening identified one DMCAD sample (GSM7976817) with a PC1 z-score marginally exceeding the predefined threshold (z = 3.02). However, because the deviation was minimal, hierarchical clustering did not indicate sample isolation, and the sample remained biologically consistent with the DMCAD group; it was retained for downstream analyses. Hierarchical clustering further confirmed the absence of distinct outlier clusters or aberrant sample behavior. Based on the combined QC metrics, no samples were excluded, and all 56 samples were included in subsequent differential expression analyses (Figure 2).

### 2.3. Differential Expression and Protein–Protein Interaction Analysis of Shared DEGs

**Figure 3:**
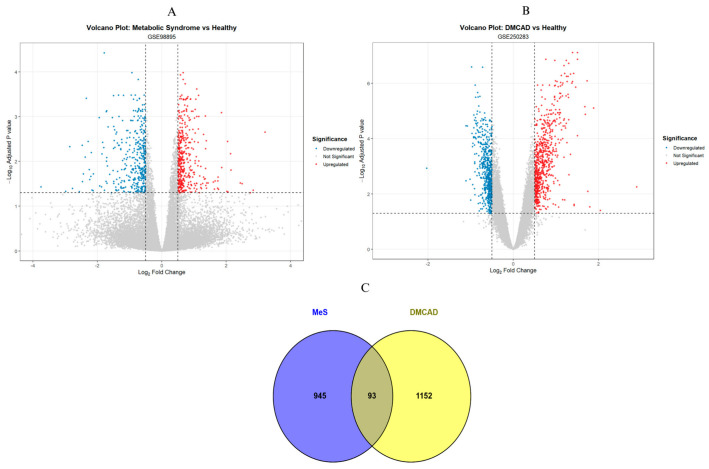
Differential gene expression analysis and identification of shared DEGs between metabolic syndrome and DMCAD. Differential expression analysis and identification of shared differentially expressed genes (DEGs) between metabolic syndrome (MeS) and diabetes mellitus-associated coronary artery disease (DMCAD). (**A**) Volcano plot showing DEGs in GSE98895 (MeS versus healthy controls). (**B**) Volcano plot showing DEGs in GSE250283 (DMCAD versus healthy controls). Significantly upregulated genes are shown in red, downregulated genes in blue, and non-significant genes in gray. (**C**) Venn diagram illustrating the overlap of DEGs between the two datasets, identifying 93 shared DEGs.

Differential expression analyses using the QC-confirmed datasets with a unified significance threshold of adjusted *p*-value < 0.05 and |log2FC| ≥ 0.5. The analysis identified 1038 DEGs in GSE98895 and 1245 DEGs in GSE250283 (DMCAD vs Healthy). Intersection analysis revealed 93 common DEGs (Figure 3). Subsequent PPI network analysis demonstrated significant interaction enrichment (92 nodes, 83 edges, PPI enrichment *p* = 4.81 × 10^−6^).

**Figure 4:**
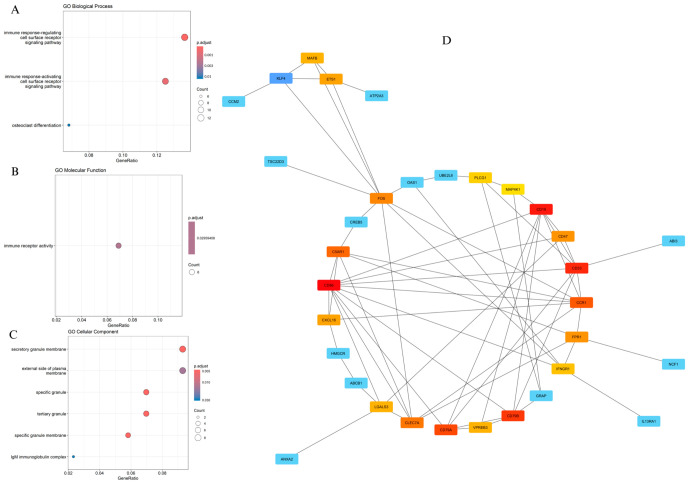
Functional enrichment and protein–protein interaction network analysis of shared DEGs. Functional characterization of the shared DEGs. (**A**–**C**) Gene Ontology (GO) enrichment analyses showing the significantly enriched Biological Process (BP), Molecular Function (MF), and Cellular Component (CC) categories, respectively. (**D**) Protein–protein interaction (PPI) network of the shared DEGs constructed using the STRING database and visualized in Cytoscape. The top 20 hub genes, identified using the CytoHubba MCC (Maximal Clique Centrality) algorithm, node color reflects the Maximal Clique Centrality (MCC) score, with warmer colors (red) indicating higher-ranked hub genes and cooler colors (blue) indicating lower-ranked hub genes.

Hub-gene analysis using the MCC algorithm identified *CD86*, *CD19*, *CD33*, *CD79B*, *CD79A*, *CCR1*, *C5AR1*, *CLEC7A*, *FOS*, and *FPR1* as the top-ranked hub genes. Several previously identified inflammatory genes, including *CCR1*, *C5AR1*, *FPR1*, *CXCL16*, and *LGALS3*, remained prominent nodes within the network, confirming the robustness of the findings following quality-control-based reanalysis (Figure 4).

GO Molecular Function analysis identified immune receptor activity as the most significantly enriched molecular function term, involving *C5AR1*, *CCR1*, *FPR1*, *IFNGR1*, *IL13RA1*, and *LILRA3*. Cellular Component enrichment demonstrated significant overrepresentation of specific granule membrane, tertiary granule, secretory granule membrane, and external side of plasma membrane categories, indicating enrichment of genes involved in leukocyte activation and immune-cell signaling.

### 2.4. Hallmark Pathway Enrichment Reveals Shared Inflammatory Signatures in MeS and DMCAD

**Figure 5:**
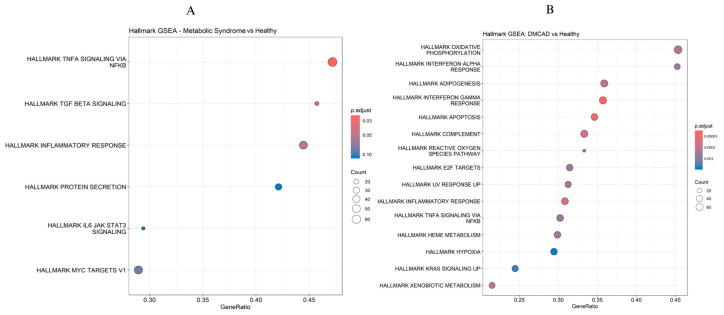
Gene set enrichment analysis of metabolic syndrome and DMCAD. Hallmark gene set enrichment analysis (GSEA) of metabolic syndrome (MeS) and diabetes mellitus-associated coronary artery disease (DMCAD). (**A**) Significantly enriched Hallmark pathways identified in GSE98895 (MeS versus healthy controls). (**B**) Significantly enriched Hallmark pathways identified in GSE250283 (DMCAD versus healthy controls). Bubble size represents the number of enriched genes, whereas color intensity indicates the adjusted *p*-value.

Hallmark GSEA identified multiple significantly enriched pathways in both Metabolic Syndrome and DMCAD. In the MeS dataset, the most significantly enriched pathways included TNFα signaling via NF-κB, inflammatory response, IL6-JAK-STAT3 signaling, TGF-β signaling, protein secretion, and MYC targets V1. Among these, TNFα signaling via NF-κB and inflammatory response exhibited the strongest enrichment, indicating activation of pro-inflammatory signaling networks during the early stages of metabolic dysfunction (Figure 5).

In the DMCAD dataset, a broader spectrum of significantly enriched pathways was observed. In addition to inflammatory response and TNFα signaling via NF-κB, pathways associated with interferon-α response, interferon-γ response, complement activation, reactive oxygen species pathway, apoptosis, hypoxia, oxidative phosphorylation, heme metabolism, adipogenesis, and E2F targets were significantly enriched. Oxidative phosphorylation and interferon signaling pathways showed particularly strong enrichment, suggesting extensive metabolic reprogramming and immune activation in patients with diabetic coronary artery disease.

Notably, inflammatory response and TNFα signaling via NF-κB were enriched in both datasets, indicating that chronic inflammation represents a common molecular feature linking Metabolic Syndrome with cardiovascular complications. The greater number and diversity of enriched pathways identified in DMCAD suggest progression from a predominantly inflammatory state in MeS toward a more complex disease phenotype characterized by immune dysregulation, oxidative stress, mitochondrial dysfunction, and tissue remodeling.

### 2.5. WGCNA Identified a DMCAD-Associated Blue Module Enriched in Innate Immune and Inflammatory Pathways

**Figure 6:**
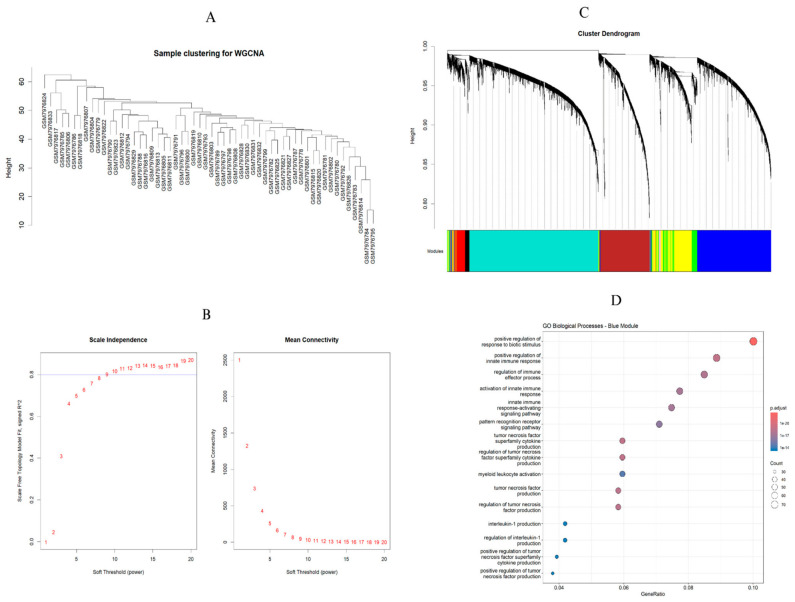
Hallmark pathway enrichment and WGCNA analysis in DMCAD. Weighted gene co-expression network analysis (WGCNA) of the GSE250283 dataset. (**A**) Sample clustering dendrogram used for network construction. (**B**) Scale-free topology fit index and mean connectivity plots used to determine the optimal soft-thresholding power. (**C**) Gene clustering dendrogram with module color assignments identified by dynamic tree cutting. (**D**) Gene Ontology Biological Process enrichment analysis of the DMCAD-associated blue module, highlighting innate immune and inflammatory pathways.

WGCNA identified eight co-expression modules from the 5000 most variable genes. Module–trait correlation analysis demonstrated that the blue module exhibited the strongest positive association with diabetic coronary artery disease (DMCAD) (r = 0.698, *p* = 2.27 × 10^−9^), indicating that genes within this module were highly associated with the DMCAD phenotype (Figure 6C). Functional enrichment analysis of the blue module revealed significant enrichment of innate immune and inflammatory processes, supporting its biological relevance to disease pathogenesis (Figure 6D). In contrast, the red module showed a significant negative correlation with DMCAD (r = −0.657, *p* = 3.92 × 10^−8^).

The Blue module contained 1165 genes and was selected for downstream functional characterization. GO-BP enrichment analysis demonstrated significant enrichment of innate immune and inflammatory pathways, including positive regulation of innate immune response, activation of innate immune response, innate immune response-activating signaling pathway, pattern recognition receptor signaling pathway, regulation of tumor necrosis factor (TNF) production, positive regulation of interleukin-1 production, and myeloid leukocyte activation. These findings indicate extensive activation of innate immune signaling networks in DMCAD.

To identify central genes within this disease-associated module, intramodular hub-gene analysis was performed based on module membership (MM), which measures the correlation between each gene’s expression profile and the corresponding module eigengene.

The top-ranking hub genes included *HNMT*, *MS4A6A*, *TYROBP*, *PEA15*, *FGL2*, *ALDH2*, *KCTD12*, *RNASE2*, *LMO2*, *MNDA*, *LILRA5*, *GRN*, *S100A12*, and *CD86* among the top-ranking blue module hub genes. Notably, CD86 was also identified as a hub gene in the protein–protein interaction network, providing independent validation of its central role in disease-associated transcriptional networks (Figure 6).

### 2.6. External Transcriptomic Validation

Gene Set Enrichment Analysis (GSEA) was performed using the ranked gene list derived from the validation dataset (GSE281291). Among the Hallmark gene sets, only the HALLMARK_UNFOLDED_PROTEIN_RESPONSE pathway demonstrated significant enrichment, suggesting the involvement of endoplasmic reticulum (ER) stress-related mechanisms in metabolic dysfunction.

To further evaluate the robustness of the identified candidate biomarkers, genes obtained from multiple analytical approaches were compared. Cross-comparison between the validation dataset and hub genes identified from the WGCNA blue module revealed one common gene, *LILRA5*. Comparison between the validation dataset and CytoHubba-derived hub genes identified *CD33* as a shared gene. Additionally, overlap between WGCNA hub genes and CytoHubba hub genes identified *CD86* as a common candidate.

Based on the integration of PPI, WGCNA, and external transcriptomic validation, *LILRA5*, *CD33*, and *CD86* were prioritized as key candidate biomarkers.

### 2.7. Computational Identification of miRNAs Associated with MeS and DMCAD

Following topological filtering (degree ≥ 2 and betweenness centrality ≥ 2), a refined miRNA–gene interaction network was obtained. Network analysis identified ETS1 as the most connected gene hub (degree = 13, betweenness = 29.70), followed by KLF4 (degree = 12, betweenness = 24.39), FOS (degree = 12, betweenness = 18.57), CD47 (degree = 10, betweenness = 10.85), and PLCG1 (degree = 10, betweenness = 11.85). Additional immune-related genes retained in the network included IFNGR1, MAFB, LGALS3, FPR1, CXCL16, and CD33.

**Figure 7:**
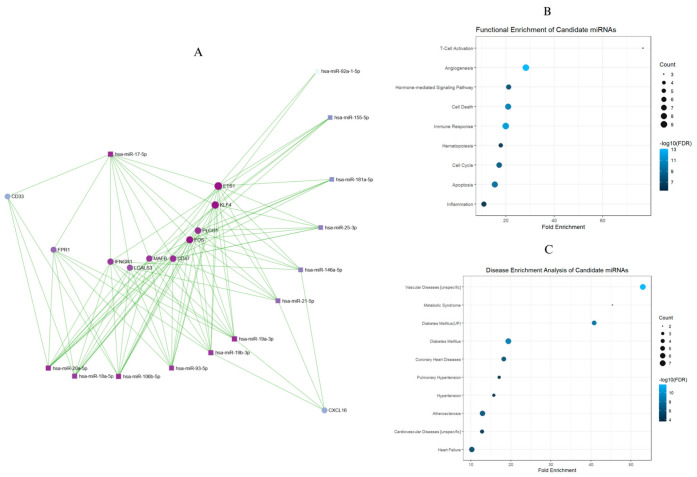
Candidate miRNA regulatory network and TAM 2.0 enrichment analysis. miRNA-mediated regulatory network and enrichment analysis of candidate miRNAs associated with MeS and DMCAD. (**A**) Filtered miRNA–gene interaction network generated using miRNet 2.0. *ETS1*, *KLF4*, and *FOS* emerged as major regulatory hubs, while members of the miR-17/92 family and miR-146a-5p represented the most connected miRNAs. (**B**) TAM 2.0 functional enrichment analysis showing significant associations with angiogenesis, immune response, apoptosis, cell-cycle regulation, and inflammation. (**C**) TAM 2.0 disease enrichment analysis demonstrating significant enrichment of cardiometabolic disease categories, including metabolic syndrome, diabetes mellitus, atherosclerosis, coronary heart disease, heart failure, and vascular diseases.

Among the miRNAs, hsa-miR-17-5p emerged as the most connected regulatory miRNA (degree = 10, betweenness = 12.79). Other highly connected miRNAs included hsa-miR-19a-3p (degree = 10), hsa-miR-20a-5p (degree = 10), hsa-miR-106b-5p (degree = 10), hsa-miR-18a-5p (degree = 9), hsa-miR-19b-3p (degree = 9), hsa-miR-93-5p (degree = 9), and hsa-miR-146a-5p. The central localization of *ETS1*, *KLF4*, and *FOS* within the network suggests their potential role as major transcriptional regulators, whereas the identified miRNAs may contribute to post-transcriptional regulation of inflammatory and metabolic pathways associated with metabolic syndrome and coronary artery disease (Figure 7A).

### 2.8. Functional and Disease Enrichment of Candidate miRNAs Associated with MeS and DMCAD 

TAM 2.0 disease enrichment analysis of the candidate miRNAs revealed significant associations with multiple cardiometabolic disorders. The most significantly enriched disease categories included vascular diseases (FDR = 1.60 × 10^−12^), diabetes mellitus (FDR = 4.91 × 10^−9^), atherosclerosis (FDR = 1.32 × 10^−6^), coronary heart disease (FDR = 1.94 × 10^−6^), and heart failure (FDR = 5.33 × 10^−6^). Notably, metabolic syndrome was also significantly enriched (FDR = 1.11 × 10^−4^), supporting the biological relevance of the identified miRNA regulatory network in cardiometabolic disease pathogenesis.

Functional enrichment analysis of the candidate miRNAs using TAM 2.0 revealed significant enrichment of several biological processes associated with immune regulation, vascular remodeling, and cardiometabolic disease. The most significantly enriched functions included angiogenesis (FDR = 8.28 × 10^−14^), immune response (FDR = 2.41 × 10^−12^), cell death (FDR = 9.39 × 10^−11^), apoptosis (FDR = 1.20 × 10^−9^), and cell cycle regulation (FDR = 1.12 × 10^−8^). Additional enrichment was observed for hormone-mediated signaling pathways, T-cell activation, hematopoiesis, and inflammation (Figure 7B,C). These findings suggest that the identified miRNAs participate in immune-inflammatory, metabolic, and vascular processes implicated in the progression of metabolic syndrome and coronary artery disease (Figure 7B).

## 3. Discussion

The present study employed an integrative bioinformatics framework to investigate the molecular mechanisms linking metabolic syndrome (MeS) with diabetes mellitus-associated coronary artery disease (DMCAD). By combining differential expression analysis, functional enrichment, protein–protein interaction (PPI) network analysis, weighted gene co-expression network analysis (WGCNA), external transcriptomic validation, and miRNA regulatory network analysis, we identified a common immuno-metabolic signature shared between both conditions. Collectively, the findings indicate that chronic immune activation, inflammatory signaling, oxidative stress, and post-transcriptional regulatory mechanisms constitute key molecular processes underlying the progression from metabolic dysfunction to cardiovascular complications.

A major finding of this study was the consistent enrichment of immune and inflammatory pathways across all analytical approaches. Functional enrichment analysis of the shared differentially expressed genes revealed significant overrepresentation of chemotaxis, immune receptor activity, complement receptor signaling, cytokine-mediated pathways, and leukocyte activation. These findings are consistent with the current understanding that metabolic syndrome is characterized by a state of chronic low-grade inflammation, often referred to as “metainflammation,” which contributes to insulin resistance, endothelial dysfunction, and atherosclerotic progression [6,7]. The identification of immune response-regulating and immune response-activating cell-surface receptor signaling pathways further suggests that dysregulated communication between immune cells may represent a common molecular mechanism linking metabolic abnormalities with cardiovascular disease.

The hub genes identified through PPI network analysis provide additional insight into the inflammatory processes driving disease progression. Among the top-ranked genes, *CCR1*, *C5AR1*, *FPR1*, *CXCL16*, *CD86*, and *CD33* have established roles in innate and adaptive immune responses. *CCR1* is a chemokine receptor involved in monocyte and leukocyte recruitment to sites of inflammation, whereas *C5AR1* mediates complement-dependent inflammatory signaling and has been implicated in atherosclerotic lesion development. *FPR1* contributes to neutrophil activation and migration, while *CXCL16* functions as both a chemokine and scavenger receptor that promotes immune-cell recruitment and vascular inflammation. *CD86* serves as a critical co-stimulatory molecule for T-cell activation and antigen presentation, and *CD33* is involved in the regulation of myeloid-cell activation and innate immune responses. The simultaneous identification of these genes supports the notion that persistent activation of both innate and adaptive immune pathways contributes to cardiometabolic disease progression.

The Hallmark GSEA results further strengthened this inflammatory model by demonstrating enrichment of TNFα signaling via NF-κB, inflammatory response, and IL6-JAK-STAT3 signaling pathways in both MeS and DMCAD. These pathways are well-recognized mediators of obesity-associated inflammation and insulin resistance [8]. NF-κB activation promotes the production of pro-inflammatory cytokines and chemokines, resulting in sustained leukocyte recruitment and vascular injury. Similarly, activation of IL6-JAK-STAT3 signaling has been associated with impaired insulin signaling, endothelial dysfunction, and chronic inflammation. The presence of these pathways in both conditions suggests that inflammatory signaling constitutes a common molecular foundation that persists throughout disease progression.

Notably, DMCAD exhibited a broader spectrum of enriched pathways compared with MeS, including interferon-α response, interferon-γ response, complement activation, reactive oxygen species (ROS) pathways, hypoxia, apoptosis, oxidative phosphorylation, and adipogenesis. These findings suggest that progression from metabolic syndrome to coronary artery disease involves the accumulation of additional pathogenic processes beyond inflammation alone [9]. Increased oxidative stress and mitochondrial dysfunction have been widely implicated in endothelial injury and atherosclerotic plaque formation, while chronic activation of interferon signaling can amplify inflammatory responses and vascular damage. Therefore, our results support a model in which metabolic syndrome initially establishes a pro-inflammatory environment that subsequently evolves into a more complex state characterized by immune dysregulation, oxidative stress, and metabolic remodeling.

The WGCNA analysis provided independent validation of the inflammatory mechanisms identified by differential expression and enrichment analyses. The DMCAD-associated blue module demonstrated strong positive correlations with disease status and was significantly enriched for innate immune activation, pattern-recognition receptor signaling, TNF production, IL-1 production, and myeloid-cell activation pathways. These observations reinforce the concept that innate immune dysregulation is a central feature of diabetic coronary artery disease. Several highly connected genes within the blue module, including *TYROBP*, *S100A12*, *LILRA5*, *MS4A6A*, and *FGL2*, have previously been implicated in inflammatory and cardiovascular disorders. *TYROBP* functions as an adaptor molecule involved in macrophage activation and inflammatory signaling [10], whereas *S100A12* is an established mediator of vascular inflammation and cardiovascular risk [11]. *LILRA5* [12] and *MS4A6A* [13] contribute to myeloid-cell activation, while *FGL2* has been linked to inflammatory and thrombotic responses [14]. Importantly, *CD86* was identified as a hub gene in both the PPI and WGCNA analyses, highlighting its potential role as a central regulator of immune activation in cardiometabolic disease.

External validation using an independent type 2 diabetes mellitus dataset identified *CD86*, *CD33*, and *LILRA5* as high-confidence candidate biomarkers. Although the validation cohort represented T2DM rather than metabolic syndrome, the close pathophysiological relationship between these conditions supports their suitability for evaluating the robustness of candidate genes. Interestingly, GSEA of the validation dataset identified significant enrichment of the unfolded protein response pathway, suggesting that endoplasmic reticulum (ER) stress may contribute to the molecular mechanisms identified in this study. ER stress has emerged as an important mediator linking nutrient excess, lipid accumulation, insulin resistance, and chronic inflammation. Persistent activation of the unfolded protein response can promote inflammatory cytokine production and immune-cell dysfunction, thereby contributing to both metabolic and cardiovascular pathology. The convergence of ER stress-related pathways with immune-associated biomarkers such as *CD86*, *CD33*, and *LILRA5* suggests that metabolic stress and immune activation may cooperate to drive disease progression.

To further investigate regulatory mechanisms underlying these transcriptional alterations, a miRNA–gene interaction network was constructed. Network analysis identified *ETS1*, *KLF4*, and *FOS* as central transcriptional regulators. *ETS1* is involved in immune-cell differentiation and inflammatory gene expression, *KLF4* regulates macrophage polarization and endothelial homeostasis, and *FOS* functions as a component of the AP-1 transcription factor complex that mediates responses to cytokines, oxidative stress, and metabolic stimuli. The central positioning of these transcription factors suggests that they coordinate major inflammatory and metabolic transcriptional programs associated with cardiometabolic disease [15].

Among the candidate miRNAs, miR-17-5p, miR-20a-5p, miR-106b-5p, and miR-146a-5p exhibited the highest network connectivity. Notably, miR-17-5p, miR-20a-5p, and miR-106b-5p belong to the miR-17 family and have been implicated in immune regulation, cell proliferation, insulin resistance, and atherosclerotic progression. In contrast, miR-146a is widely recognized as a negative regulator of inflammatory signaling and NF-κB activation [16]. The identification of these miRNAs suggests the existence of a complex post-transcriptional regulatory network that modulates inflammatory and metabolic responses during disease progression. Furthermore, TAM 2.0 enrichment analysis demonstrated significant associations with angiogenesis, immune response, apoptosis, vascular disease, diabetes mellitus, atherosclerosis, coronary heart disease, and metabolic syndrome, further supporting their biological relevance.

Taken together, the present findings support a model in which chronic immune activation serves as the central molecular bridge linking metabolic syndrome to coronary artery disease. Persistent activation of inflammatory signaling pathways, chemokine and complement receptors, innate immune networks, oxidative stress responses, and ER stress-related mechanisms appears to drive disease progression. Simultaneously, transcription factors and regulatory miRNAs provide additional layers of molecular control that may influence the transition from metabolic dysfunction to cardiovascular complications.

## 4. Methods

### 4.1. General Workflow

**Figure 8:**
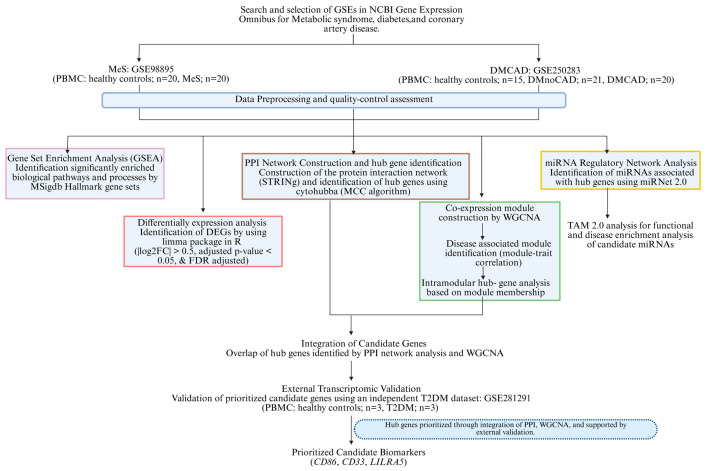
Overall workflow of the integrative bioinformatics analysis.

The overall analytical workflow of this study is illustrated (Figure 8). Briefly, transcriptomic datasets associated with metabolic syndrome (MeS) and diabetic coronary artery disease (DMCAD) were retrieved from the Gene Expression Omnibus (GEO) database. Following quality-control assessment and data preprocessing, differential expression analysis was performed to identify disease-associated genes. Functional enrichment analyses were conducted to explore the biological processes and pathways associated with the identified genes. Protein–protein interaction (PPI) network analysis and cytoHubba were subsequently employed to prioritize hub genes. To further strengthen candidate gene identification, weighted gene co-expression network analysis (WGCNA) was performed on DMCAD dataset to identify DMCAD disease-associated gene modules. Finally, the resulting candidate genes were evaluated using an independent external validation dataset, and overlapping genes across the differential expression, network, and validation analyses were identified as robust candidate biomarkers associated with DMCAD.

Schematic overview of the analytical workflow used to investigate shared immuno-metabolic mechanisms between metabolic syndrome (MeS) and diabetes mellitus-associated coronary artery disease (DMCAD). Publicly available PBMC transcriptomic datasets (GSE98895 and GSE250283) were subjected to quality-control assessment, differential expression analysis, functional enrichment analysis, protein–protein interaction (PPI) network construction. Hub genes identified by PPI network analysis and WGCNA were integrated and subsequently evaluated using an independent validation dataset (GSE281291), while GSEA was used to identify enriched biological pathways. TAM 2.0 was used for functional and disease enrichment analysis of candidate miRNAs.

### 4.2. Gene Expression Profile Data Collection

Gene Expression Omnibus (GEO) database https://www.ncbi.nlm.nih.gov/geo/ (assessed on 5 June 2026)was used to download Expression profiling datasets for metabolic syndrome (MeS) and diabetes mellitus with coronary artery disease (DMCAD). The search terms utilized were “metabolic syndrome”, “diabetes”, “coronary artery disease”, “inflammation” and “Homo sapiens”, while applying the “expression profiling by array” filter. The inclusion criteria required datasets containing RNA expression profiles generated from peripheral blood mononuclear cells (PBMCs).

GSE98895 was submitted in 2017 by the IRCCS Ospedale Oncologico di Bari and contains PBMC gene expression profiles from healthy controls and patients with metabolic syndrome, aimed at identifying transcriptomic alterations associated with the disease. The sample selection used for analysis included healthy controls (*n* = 20) and MeS cases (*n* = 20) [17].

GSE250283 was submitted in 2023 by the National Institutes of Health Philippines and includes whole-transcriptome PBMC profiles from Filipino individuals with T2DM, with and without coronary artery disease (CAD), generated using Illumina and Affymetrix microarray platforms (Affymetrix Inc., Santa Clara, CA, USA). For this study, the GPL28098 platform containing Illumina HumanHT-12 V4.0 expression data was selected for analysis. The sample selection used for analysis included DMnoCAD (*n* = 21) and DMCAD (*n* = 20) groups. In addition, healthy controls (*n* = 15) present in the dataset were used to identify DEGs in comparison with both disease groups [18].

GSE281291 was submitted in 2024 by Chen and Long and is an independent PBMC transcriptomic dataset comprising healthy controls (*n* = 3) and patients with type 2 diabetes mellitus (T2DM; *n* = 3), generated using the GPL26963 Arraystar Human lncRNA V5 microarray platform (Arraystar Inc., Rockville, MD, USA). Although the platform contains both lncRNA and protein-coding transcripts, only protein-coding genes were retained for analysis. The dataset was used as an external validation cohort. Differential expression and pathway enrichment analyses were performed to assess the reproducibility of candidate biomarkers identified through differential expression analysis, weighted gene co-expression network analysis (WGCNA), and protein–protein interaction (PPI) network analysis [19].

### 4.3. Dataset-Level Quality Assessment and Quality Control

Quality control (QC) was performed prior to differential expression analysis to evaluate sample comparability, detect technical variability, and identify potential outlier samples. For each dataset, normalized expression matrices were subjected to multiple complementary QC procedures, including density distribution analysis, boxplot assessment, principal component analysis (PCA), and hierarchical clustering in R (version 4.5.3).

Density plots and boxplots were generated to assess the overall distribution of expression values across samples and to verify the effectiveness of normalization procedures. PCA was performed using the 5000 most variable genes (ranked by variance across samples) to evaluate global expression patterns and sample relationships. Samples with PC1 or PC2 coordinates exceeding ±3 standard deviations from the dataset mean were predefined as potential outliers.

To further assess sample similarity, hierarchical clustering was conducted using Euclidean distance and complete-linkage clustering. Samples identified as potential outliers by PCA were subsequently evaluated within the hierarchical clustering dendrogram. Samples were considered for exclusion only when supported by multiple QC metrics, including PCA separation, clustering behavior, and abnormal expression distributions. All exclusion decisions were documented and reported transparently.

For the GSE250283 dataset, QC was performed on 56 peripheral blood mononuclear cell (PBMC) samples, comprising 15 healthy controls, 21 diabetes mellitus patients without coronary artery disease (DMnoCAD), and 20 diabetes mellitus patients with coronary artery disease (DMCAD). The same QC workflow was applied to all datasets included in the study to ensure consistency and comparability across analyses.

### 4.4. PPI Network Construction and Hub Gene Identification

The DEG lists identified using the limma package (version 3.66.0) and shared between MeS and DMCAD were uploaded to the STRING database (version 12.0) for protein–protein interaction (PPI) network construction. The minimum required interaction score was set to 0.400 (medium confidence), and the network was generated using only the query proteins without adding additional interactors. The generated PPI network was imported into Cytoscape (version 3.10.4) for network visualization and topological analysis. Hub proteins were identified using the CytoHubba plugin in Cytoscape, which ranks nodes according to their topological importance within the interaction network. Among the 11 available algorithms, Maximal Clique Centrality (MCC) was selected due to its reported superior sensitivity and reliability in identifying essential proteins within densely interconnected biological subnetworks. The top 10 ranked proteins based on MCC scores were selected as hub proteins for downstream analyses.

### 4.5. Gene Set Enrichment Analysis (GSEA)

To identify coordinated biological pathways associated with Metabolic Syndrome (MeS) and diabetic coronary artery disease (DMCAD), Gene Set Enrichment Analysis (GSEA) was performed using the ClusterProfiler package (version 4.18.4) in R. All genes were ranked according to their moderated t-statistics obtained from the limma differential expression analysis, thereby avoiding arbitrary DEG cutoffs. Hallmark gene sets from the Molecular Signatures Database (MSigDB Hallmark Collection, H category) were retrieved using the msigdbr package (version 26.1.0). Enrichment analysis was performed using the GSEA algorithm implemented in ClusterProfiler, and pathways with adjusted *p*-values below 0.05 were considered significantly enriched. The results were visualized using Hallmark GSEA dot plots, where dot size represents the number of genes contributing to each pathway and color indicates the adjusted *p*-value.

### 4.6. Weighted Gene Co-Expression Network Analysis (WGCNA)

To identify disease-associated gene co-expression modules, Weighted Gene Co-expression Network Analysis (WGCNA) was performed using the WGCNA package in R (version 1.74). The normalized expression matrix from GSE250283 was used as input. To reduce noise and computational burden, the top 5000 genes with the highest variance across samples were selected for network construction. Sample quality was assessed using hierarchical clustering, and no outlier samples were identified. A soft-thresholding power of 9 was selected based on the scale-free topology criterion (R^2^ > 0.8). An adjacency matrix was constructed using a signed network approach and subsequently transformed into a Topological Overlap Matrix (TOM). Genes were hierarchically clustered based on TOM dissimilarity, and modules were identified using the dynamic tree-cutting algorithm with a minimum module size of 30 genes. Module eigengenes were calculated and correlated with clinical traits (Healthy, DMnoCAD, and DMCAD) to identify disease-associated modules. Functional enrichment analysis of significant modules was performed using Gene Ontology Biological Process (GO-BP) enrichment analysis. Intramodular hub genes were identified based on module membership (MM), representing the correlation between individual gene expression profiles and the corresponding module eigengene.

### 4.7. External Transcriptomic Validation of Hub Genes

To evaluate the robustness of the identified hub genes, an independent transcriptomic dataset, GSE281291, was employed for external validation. This dataset comprises peripheral blood mononuclear cell (PBMC) samples from patients with type 2 diabetes mellitus (T2DM; *n* = 3) and healthy controls (*n* = 3) profiled using the GPL26963 Arraystar Human lncRNA V5 microarray platform.

Although GSE281291 was generated in a T2DM cohort rather than a metabolic syndrome cohort, it was selected because metabolic syndrome is a major precursor of insulin resistance, type 2 diabetes mellitus, and cardiovascular disease. Consequently, both conditions share several underlying pathogenic mechanisms, including chronic low-grade inflammation, immune dysregulation, oxidative stress, and metabolic dysfunction. Therefore, genes consistently identified across MeS and T2DM datasets may represent biologically relevant molecular signatures associated with cardiometabolic disease progression.

The GPL26963 platform contains both lncRNA and protein-coding transcripts. For external validation, only transcripts annotated as “protein_coding” were retained to enable direct comparison with the protein-coding hub genes identified in the discovery cohort. Differential expression analysis was performed. Gene Set Enrichment Analysis (GSEA) was conducted using ranked protein-coding genes and Hallmark gene sets obtained from the Molecular Signatures Database (MSigDB). Candidate genes identified through differential expression analysis, weighted gene co-expression network analysis (WGCNA), and protein-protein interaction (PPI) network analysis were subsequently compared with genes detected in the external validation dataset. Genes consistently identified across multiple analytical approaches were considered high-confidence candidate biomarkers.

### 4.8. Computational Identification of miRNAs Associated with MeS and DMCAD and miRNA–Gene Regulatory Network Construction

To investigate potential post-transcriptional regulatory mechanisms underlying the identified hub genes, a miRNA-gene interaction network was constructed using the miRNet 2.0 platform (https://www.mirnet.ca/, accessed on 20 June 2026). Candidate genes identified through differential expression analysis, weighted gene co-expression network analysis (WGCNA), and protein–protein interaction (PPI) network analysis were uploaded into miRNet. Experimentally validated and predicted miRNA-target interactions were retrieved from integrated miRNet databases.

The generated network was analyzed using the Network Viewer module. Topological properties, including degree centrality and betweenness centrality, were calculated for all nodes. Degree centrality represents the number of direct interactions connected to a node, whereas betweenness centrality measures the extent to which a node acts as a bridge connecting different regions of the network. To identify key regulatory elements and reduce network complexity, nodes with degree and betweenness centrality values greater than or equal to 2 were retained for downstream analysis. The filtered network was visualized using the concentric-circle layout, where highly connected nodes were positioned toward the center of the network and less connected nodes toward the periphery. Genes and miRNAs exhibiting high degree and betweenness values were considered potential regulatory hubs.

### 4.9. miRNA Functional and Disease Enrichment Analysis

To investigate the biological significance of the candidate miRNAs identified from the miRNA–gene interaction network, functional and disease enrichment analyses were performed using the TAM 2.0 (Tool for Annotations of Human MicroRNAs) database http://www.lirmed.com/tam2/, accessed on 20 June 2026). Candidate miRNAs were selected from the miRNet-derived Gene2miRNA network based on network topology parameters, including degree and betweenness centrality. MiRNAs with degree ≥ 5 and betweenness centrality ≥ 1 were retained for downstream analysis.

The selected miRNAs were submitted to TAM 2.0 for over-representation analysis using its manually curated miRNA-function and miRNA-disease annotation database. Enrichment significance was assessed using hypergeometric testing, and *p*-values were adjusted for multiple testing using the false discovery rate (FDR) method. Terms with FDR-adjusted *p*-values < 0.05 were considered statistically significant.

To facilitate biological interpretation, the top enriched functional categories and cardiometabolic disease-related terms were visualized in R (version 4.5.3) using bubble plots [20].

## 5. Conclusions

This integrative bioinformatics study identified shared immuno-metabolic and inflammatory transcriptional alterations between metabolic syndrome (MeS) and diabetes mellitus-associated coronary artery disease (DMCAD). Comparative analyses revealed a substantial overlap of differentially expressed genes involved in immune activation, leukocyte recruitment, cytokine signaling, complement activation, and vascular inflammation, supporting the concept that chronic low-grade inflammation represents a key molecular mechanism linking metabolic dysfunction to cardiovascular complications.

Functional enrichment analyses consistently highlighted inflammatory response, TNFα/NF-κB signaling, IL6-JAK-STAT3 signaling, innate immune activation, and oxidative stress-related pathways. Network-based analyses identified several hub genes, including *CD86*, *CD33*, *CCR1*, *C5AR1*, *FPR1*, *CXCL16*, and *LILRA5*, suggesting an important role for immune-cell communication, inflammatory receptor signaling, and myeloid-cell activation in disease progression. WGCNA further supported the involvement of innate immune pathways and identified disease-associated co-expression modules enriched for inflammatory signaling.

Integration of miRNA regulatory analysis revealed a coordinated post-transcriptional network involving members of the miR-17/92 cluster, miR-106b-5p, miR-146a-5p, miR-21-5p, and miR-155-5p. TAM 2.0 enrichment analysis demonstrated significant associations with angiogenesis, immune response, inflammation, diabetes mellitus, atherosclerosis, coronary heart disease, heart failure, and metabolic syndrome, further supporting the biological relevance of these candidate miRNAs in cardiometabolic disease pathogenesis.

Collectively, these findings support the presence of interconnected immune-inflammatory, metabolic, and vascular regulatory networks underlying the transition from metabolic syndrome to cardiovascular complications. The identified hub genes and miRNAs may serve as potential biomarkers and therapeutic targets associated with inflammation-driven cardiometabolic disease progression. However, further experimental validation and longitudinal clinical studies are required to confirm their mechanistic roles and translational utility.

## 6. Limitations

The analyses were based on publicly available PBMC transcriptomic datasets generated from independent cohorts and different microarray platforms, which may introduce population- and platform-specific variability. Because each dataset was analyzed independently and only biological signatures were compared, batch correction across studies was not performed. In addition, limited clinical metadata prevented assessment of potential confounding effects of medications, disease duration, and other patient-specific factors. Ethnic and geographic differences among cohorts may also influence transcriptomic profiles and affect cross-cohort comparability. Furthermore, the external validation dataset comprised a relatively small sample size and represented an independent Type 2 diabetes mellitus (T2DM) cohort rather than a direct DMCAD cohort, reflecting the limited availability of suitable public PBMC transcriptomic datasets. Therefore, the external validation should be considered supportive rather than definitive. Finally, the findings are based on computational analyses and require validation in larger independent cohorts, as well as experimental and clinical studies, to confirm their biological significance and translational potential.

## Data Availability

No new data were created or analyzed in this study. Data sharing is not applicable to this article.
